# Outcome of assisted reproductive technology in women aged 40 years and older

**Published:** 2011

**Authors:** Abbas Aflatoonian, Maryam Eftekhar, Farnaz Mohammadian, Fariba Yousefnejad

**Affiliations:** 1 Department of Obstetrics and Gynecology, Research and Clinical Center for Infertility, Shahid Sadoughi University of Medical Sciences, Yazd, Iran.; 2 Department of Obstetrics and Gynecology, Zanjan University of Medical Sciences, Zanjan, Iran.

**Keywords:** *Advanced age*, *Assisted reproductive technology*, *Pregnancy rate*, *Live birth*

## Abstract

**Background:**

Human fertility has been declined all over the world. Advanced women’s age is one of the most important factors in determining the success of reproduction and ageing has negative impact on ART outcome and advanced female age decreases the chance of live birth rates achieved using ART, especially after 40 years of age.

**Objective:**

To evaluate ART outcomes regarding to pregnancy, abortion, cycle cancellation and live birth rates in women 40 years and older.

**Materials and Methods:**

A retrospective study was performed on three hundred-thirteen women undergoing ART cycles in the Madar Hospital in Yazd. Women with age ≥ 40 years who indicated for ART enrolled the study regardless of the infertility type or etiology. In this study, we used data from IVF or ICSI cycles using fresh embryo transfer. Follow up was performed in regard to pregnancy, abortion, cycle cancellation and live birth rates.

**Results:**

The mean age of women was 41.87±1.97 years. Chemical pregnancy rate was 8.6% (27/313) per cycle. Clinical pregnancy rate was 3.8% (12/313) per cycle. Spontaneous abortion was observed in 63% (17/27) of patients with positive pregnancy test. The overall cancellation rate was 23.3% per oocytes retrieval. The overall live birth rate per cycle for all women who initiated an ART cycle at age ≥40 years was 3.2% (10/313) that eight of those women were under 42 years old**.**

**Conclusion:**

Based on our results, we suggest that women with age 42 years and above should be advised to use other options, including oocyte donation or adoption.

## Introduction

Human fertility has been declined all over the world, including both developed and developing countries. It is generally believed that more than 70 million couples suffer from infertility worldwide. Several factors influence fertility decline that some of these factors are unknown, although advanced women’s age is one of the most important factors in determining the success of reproduction in natural fertility or after artificial insemination (1-6). 

A major health problem is women who delay child bearing until late reproductive years and 50% of them will have some difficulty in their attempt to have children (1, 7-8). The female reproductive ageing is characterized by obvious decline in ovarian germ cells supply, decreased in oocyte quality and ultimately leading to ovarian reproductive failure. These usually start at 32 years and a marked decline in fecundity and fertility rates occurs in women older than 35 years and accelerates in women≥40 years old. 

In addition of decreased fecundity in older women, there is an increase in the spontaneous abortion rates in these women (7-14). Use of assisted reproductive technology (ART) has steadily increased during the past two decades and it has been shown 13% to 16% increase in the use of ART services. It was reported that 19% of all women using ART are aged 40 years and older (7, 13, 15). 

Ageing has negative impact on ART outcome and advanced female age decreases the chance of live birth rates achieved using ART, especially after 40 years of age. Even some IVF centers refuse women over the age of 40 years because of lower success of ART outcome. ART is expensive, time-consuming and stressful procedure, so infertile couples should be aware of expected chance of achieving pregnancy before deciding to start ART treatment (6, 8-9, 13, 15).

The aim of this study was the assessment of ART outcomes regarding; pregnancy, abortion, cycle cancellation and live birth rates in women 40 years and older.

## Materials and methods

A retrospective study was performed on 313 women undergoing ART cycles with age≥ 40 years in the Madar Hospital in Yazd from January 2008 to December 2010. In our study we used data from ART cycles using fresh embryo transfer regardless of the infertility type or etiology. Donor or surrogate cycles, ZIFT or GIFT and freeze embryo transfer were excluded from the study. 

Controlled ovarian stimulation in ART cycles was done using long down-regulation or short flare-up protocols with gonadotropin-releasing hormone (GnRH) agonist and HMG/r-FSH or GnRH antagonist with HMG/r-FSH or only HMG protocols. When at least two follicles reached a mean diameter of 18 mm, using transvaginal ultrasonography, 10000 IU HCG was administrated. Oocytes pick-up was performed 34-36 hours after HCG injection and conventional IVF or ICSI were done as appropriately.

Embryo transfer was performed on the day 2 or 3 after oocytes retrieval with using a Labotect catheter (Labotect, Gottingen^, ^Germany) based on the number and quality of obtained embryos. Luteal phase support with 100 mg of intramuscular progesterone in oil (progesterone, Aburaihan Co., Tehran, Iran) or 400 mg of vaginal progesterone (Cyclogest®; Actavis, Branstaple, UK) was started from the day of oocytes pick-up and was continued until the negative pregnancy test or the end of the first trimester of pregnancy. Follow-up of ART outcomes was done based on chemical pregnancy, clinical pregnancy, abortion, cycle cancellation, ongoing pregnancy and live birth and was carried out by chart review and/or telephone communication.

Chemical pregnancy was identified with measuring serum beta-hCG levels 14 days after embryo transfer. Clinical pregnancy was considered as the observation of fetal heart activity by transvaginal ultrasonography, 3 weeks after positive beta-hCG. Spontaneous abortion was defined as loss of fetus with gestational age< 20 weeks. Cycle cancellation was identified when no embryo was transferred because of no oocyte retrieval or no obtained embryo. A live birth was defined as the birth of at least one live born after 20 weeks.

**Statistical analysis**

Statistical analysis was performed using the statistical package for the social science version 15.0 for windows (SPSS, Inc., Chicago, IL). Chi-square, nonparametric and Student's t-tests were used as appropriate. P value of less than 0.05 was considered statistically significant.

## Results

The study was performed on 313 women undergoing ART cycles. Women who enrolled the study were with a range of 40-49 years. The patients were stratified into seven groups by age. The frequency and percentage of women's age were summarized in Table I. The mean age of these women was 41.87±1.97 years. The mean number of retrieval oocytes was 3.20±2.76. The mean number of obtained embryos was 1.84±1.63 and the mean number of transferred embryos was 1.60±1.13 (Table II). 

The overall chemical pregnancy rate was 8.6% (27/313) per cycle. Clinical pregnancy rate was 3.8% (12/313) per cycle. The abortion rate was found in 63% (17/27) of patients with positive pregnancy test. The overall cancellation rate was 23.3% per oocytes retrieval (Table III). The overall live birth rate per cycle for all women who initiated an ART cycle at age≥40 years was 3.2% (10/313) that eight of those women were under 42 years old (Table III). 

**Table I:** Number and percentage of women's in different age groups

**Age (years)**	**No. of patients**	**Percentage of patients**
40	101	32.3%
41	63	20.1%
42	58	18.5%
43	28	8.9%
44	26	8.3%
45	15	4.8%
≥ 46	22	7.1%
Total	313	100%

**Table II:** Cycle response based on women's age

**Age (years)**	**No. of retrieved oocytes**	**No. of obtained embryos**	**No. of transferred embryos**
40	3.8±3.0	2.2±1.8	1.9±1.1
41	3.2±2.4	1.9±1.4	1.7±1.1
42	2.6±2.1	1.5±1.1	1.4±1.0
43	3.1±2.6	1.6±1.4	1.5±1.0
44	3.6±3.9	2.2±2.1	1.6±1.1
45	2.4±1.7	1.5±1.0	1.5±1.0
≥ 46	1.6±1.7	0.8±1.1	0.8±1.1
Total	3.2±2.7	1.8±1.6	1.6±1.1

**Table III:** ART outcomes based on women's age

**Age (years)**	**Chemical pregnancy per cycle (N%)**	**Clinical pregnancy per cycle (N%)**	**Abortion per positive pregnancy test (N%)**	**Cycle cancelation per oocyte retrieval**	**Live birth per cycle (N%)**
40	7 (6.9%)	3 (3.0%)	4 (57.1%)	17 (16.8%)	3 (3.0%)
41	11 (17.5%)	6 (9.5%)	6 (54.5%)	12 (19%)	5 (7.9%)
42	3 (5.2%)	1 (1.7%)	3 (100%)	17 (23.3%)	0 (0.0%)
43	2 (7.1%)	1 (3.6%)	1 (50%)	6 (21.4%)	1 (3.6%)
44	3 (11.5)	0 (0.0%)	3 (100%)	6 (23.1%)	0 (0.0%)
45	1 (6.1)	1 (6.7%)	0 (0.0%)	3 (20%)	1 (6.7%)
≥ 46	0 (0.0%)	0 (0.0%)	0 (0.0%)	12 (54%)	0 (0.0%)
Total	27 (8.6%)	12 (3.8%)	17 (63%)	73 (23.3%)	10 (3.2%)

## Discussion

Age of the women is the most important factor in determining pregnancy success rates in natural conception and after ART. The decline in fertility remains a complex issue for women with advanced age, although trend toward delaying childbirth continues (7, 13, 16). The overall outcome of ART in our study showed a clinical pregnancy rate of 3.8% per cycle and chance of live birth rates was 3.2% that most of them occurred in women aged lower than 42 years. 

Lass *et al* found a clinical pregnancy rate of 12.7% in women aged 40-43 years and a 3.2% ongoing pregnancy rate per cycle, although they did not report live birth in their study (4). Ron-El *et al* and Widra *et al* showed no pregnancies beyond frothy-third years (5, 17) and in our study, no pregnancy and live birth were found in women aged 45 years and older. Ernest Hung *et al* reported 2.5% live birth in women 45 years and no women older than 45 years had a child (18). Also based on previous several published studies, no pregnancy could be achieved among women aged ≥ 45 years when using autologous oocytes (4-5, 17, 19).

It appears that the negative influence of advanced age involves mainly ART outcomes including cancellation rate. In recent study, the cancellation rate was 23.3% per cycle, with a sharp rise at age 45 and above. Serour *et al* reported the cancellation rate of 16% per initiated cycle, which is similar to the 16.6% in Tsafrir *et al* study (7, 20). Klipstein *et al* showed a cancellation rate of 19.9% (13). Clearly, fetal loss is greatly impacted by maternal age and there is a significant increase in aneuploidy in older oocytes. Some studies have reported varied abortion rates with advancing age (4, 21-23). For women aged above 40 years, the singleton pregnancy loss rate was 27% (21), whereas present study determined 63.7% abortion rate per cycle. Harrison *et al* study showed higher rate of abortion (40% at 40 years and ≥83% at ≥41 years) (23). 

In our study, the live birth rate for those women who initiating ART at age ≥40 years were 3.2%. The chances of women ≥40 years for achieving pregnancy by using ART decline every year, so it is not clinically appropriate to start an ART cycle in patients 43 years and above with autologous oocytes (7) and according to our ART outcomes, this issue is confirmed in women 42 years and older. An important factor to the low live birth rate in older women is the significant impact of pregnancy loss (13). 

Other possible factors that may have detrimental effects on ART outcomes are damaged to endometrium and increased fibroid and/or endometriosis in older women (5). The results of oocyte donation studies suggested that declining uterine receptivity may contribute to the poor outcome in advanced age (12, 24). Also the older women have a limited number of good quality oocytes that have an increased risk of chromosomal anomalies and poor IVF outcomes with autologous oocytes (12, 22, 25). 

Counseling with couples is important in women with advanced age and an oocyte donation or adoption programs may be a more reasonable alternative, if applicable (22, 25). Henne *et al* have shown when there is a live birth rate < 5%, the cost of ART cycles is greatly higher than of donor cycle (26). Even some IVF centers refuse women over the age of 40 years because of the natural decline in fertility with age and the higher incidence of genetic anomalies in infants of these women (6).

## Conclusion

Based on our results, we suggest that women with age 42 years and above should be advised to use other options, including oocyte donation or adoption, although it is an important and difficult decision.
